# Investigating the Interfacial Structure of Potato Protein Microgels at the Air–Water Interface

**DOI:** 10.1021/acs.langmuir.5c04113

**Published:** 2025-12-16

**Authors:** Daisy Z. Akgonullu, Ryan Marr, Brent S. Murray, Simon D. Connell, Amin Sadeghpour, Yuan Fang, Bruce Linter, Anwesha Sarkar

**Affiliations:** † Food Colloids and Bioprocessing Group, School of Food Science and Nutrition, 4468 University of Leeds, Leeds LS2 9JT, U.K.; ‡ Molecular and Nanoscale Physics Group, School of Physics and Astronomy, University of Leeds, Leeds LS2 9JT, U.K.; § National Alternative Protein Innovation Centre (NAPIC), Leeds LS2 9JT, U.K.; ∥ PepsiCo, Valhalla, New York, NY 10595, United States; ⊥ 438290 PepsiCo International Ltd, Leicester LE4 1ET, U.K.

## Abstract

This study investigates the role of size and deformability of potato protein microgels in influencing their interfacial performance at the air–water interface. Microgels produced via a top-down method were studied across different length scales, focusing on the air–water interface. Techniques included internal structure analysis via small-angle X-ray scattering (SAXS), particle deformability and moduli studies using atomic force microscopy (AFM), and compression and deposition of Langmuir–Blodgett monolayers. It was found that microgels have the capacity to reach a jammed interfacial state similar to that of nongelled potato protein, however, compression may be required to promote their intermolecular interactions. Despite this, thicker microgel-laden interfacial layers may have greater capacity to promote steric hindrance and aid stability within foams and emulsions.

## Graphical abstract

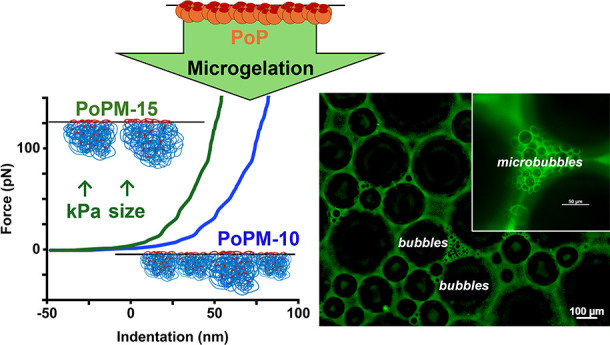

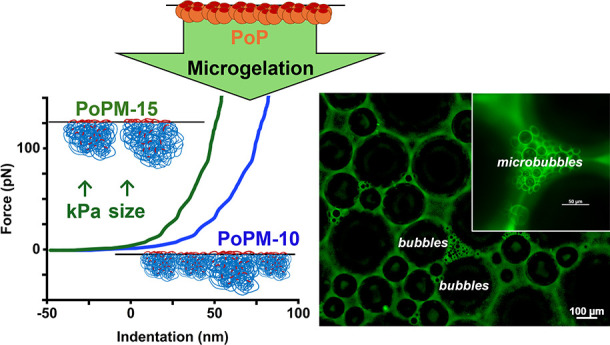

## Introduction

Microgels are deformable particles formed of cross-linked polymers, ranging in size from ∼100 nm to several microns.^1^ Their unique deformability has been widely explored as a means of tuning the bulk characteristics of solutions, since microgels are reported to interpenetrate and reach extremely high levels of particle jamming which facilitate viscoelasticity modification.^2^ Not only do microgels have the capacity to alter bulk attributes, but they also possess surface activity with high desorption energies from liquid–liquid interfaces, enabling them to act as emulsion and foam stabilizers, preventing coalescence and Ostwald ripening.^3^

Microgels are typically considered to possess a “core–corona” structure, where the core polymers form a dense mass surrounded by a periphery of more loosely cross-linked chains.^2^ However, these architectures are largely associated with microgels formed from synthetic polymers,^4^ meaning further insight is needed into the mechanisms controlling the behavior of microgels of biopolymeric origin *i*.*e*. those formed from polysaccharides or protein. These appear to have more unique characteristics which are dictated by the biopolymer from which they are fabricated.^5,6^ Furthermore, it cannot be assumed that their internal particle structure is the same as the more uniform synthetic microgels generally formed via more controlled methods of fabrication.^1^

Many recent studies have shown the importance of microgel elasticity with respect to their ability to act as emulsion stabilizers.^2,7–11^ Higher elasticity is the result of increased polymer and/or cross-linker concentration and controls the architecture of the resultant microgel particles and their capacity for deformation.^2,11,12^ Deformation of microgels may facilitate the development of a more cohesive, viscoelastic interfacial film which provides greater resilience to mechanical strain.^3,4,13^ For the formation of interfacial monolayers which effectively promote emulsion stability, it is thought that close, structured packing of stabilizing particles is a critical requirement.^14^ The viscoelastic nature of microgels may enable them to adapt their configuration against emulsion destabilization, yet it appears that there may be an optimal level of cross-linking.^15^ Beyond this, although the thickness of the monolayer may offer steric stability,^16^ particles may be restricted from forming flexible interfacial networks and instead create a system of inert, rigid aggregates of lower interfacial viscoelasticity.^17,18^ Although the role of microgel viscoelasticity in emulsion and foam stabilization is relatively well-studied in the context of synthetic and animal protein-based microgels,^19–21^ such knowledge of more sustainable, plant protein-based microgels is limited. At the same time, plant proteins tend to have more complex, aggregated structures.

Since plant-based proteins offer a more sustainable, less carbon-intensive supply of protein as compared to conventional animal-based protein sources, our previous research has explored the behavior of potato protein (PoP),^22^ a plant protein of high gelling efficiency.^23,24^ In addition to potatoes being widely cultivated,^25^ PoP may also be sourced from industry waste streams^26^ and possesses an amino acid profile sequence to that of egg protein.^27^ Thus, PoP is of great interest from industrial, nutritional and sustainability viewpoints. In our previous work, PoP microgels were prepared via thermal cross-linking of PoP solutions, followed by high-shear homogenization of the resulting bulk PoP gel.^22^ Surprisingly, although the bulk PoP gel structure showed increased bulk moduli with greater PoP concentration, the interfacial viscoelasticity (at the oil–water interface) of the corresponding microgels was lower than that of the nonmicrogelled PoP.^22^ It was hypothesized that this was related to the formation of a more clustered and less cohesive interfacial structure with the PoP microgels, which restricted the formation of lateral interactions, in contrast to the nonmicrogelled PoP that formed a cohesive viscoelastic network.^18,22^ What still remains unclear is the role of size and deformability of the PoP microgels in influencing their interfacial performance. PoP is reported to act as an excellent foam stabilizer, particularly in systems containing particles of a range of colloidal sizes, which have been suggested to act synergistically in promoting foam stability.^28^ This further highlights the need for an in-depth nanoscale investigation of the interfacial structure of PoP in microgel form to identify the role of size and moduli at a particle level, for their use in emulsion and foam stabilization.

Therefore, this study aims to detail the microgel structure and interfacial behavior of PoP microgels systematically, focusing on the air–water interface, since this greatly simplifies the methodology compared to the oil–water interface. The internal structure of the microgel biopolymer network is compared to nongelled PoP for the first time within a bulk gel via small-angle X-ray scattering (SAXS), in order to understand the temperature-dependent nanostructural changes of PoP in forming the microgels. Particle deformability and moduli at the microgel particle level was investigated using atomic force microscopy (AFM). Finally, monolayers of microgels of different size and deformability at an air–water (A-W) interface were measured using Langmuir isotherms, then transferred via Langmuir–Blodgett (L-B) deposition for subsequent AFM imaging. Microgel behavior was also compared with nonmicrogelled protein. The isotherms describe microgel behavior under compression and reveal the extent of intermolecular interactions. Imaging of the L-B monolayers with AFM aimed to provide greater insight into particle conformation and aggregation of PoP microgels of different sizes and deformability. To our knowledge, this is the first study of monolayer experiments of PoP microgels and the AFM force spectroscopy of microgel moduli, which should aid further understanding of the interfacial behavior of plant based microgels and the practical application of such materials.

## Experimental Section

### Materials

Sosa ‘Potatowhip’ potato protein, containing ∼ 90% protein, was purchased from Henley Bridge (Lewes, U.K.). Previous work by Kew et al.^29^ has confirmed that this protein is mainly composed of patatin, therefore further discussion of this material refers to the properties of patatin. Tetradecane and 4-(2-hydroxyethyl)-1-piperazineethanesulfonic acid (HEPES) buffer were obtained from Fisher Scientific U.K. Ltd. (Loughborough, U.K.). Silicon wafers were obtained from Agar Scientific Ltd. (Essex, U.K.) and all atomic force microscopy (AFM) cantilevers were sourced from Bruker U.K. Ltd. (Coventry, U.K.). All other chemicals were purchased from Fisher Scientific U.K. Ltd. (Loughborough, U.K.) and all solutions were prepared with Milli-Q water (purified using Milli-Q apparatus, Millipore Corp., Bedford, MA).

### Preparation of Potato Protein Solutions

A solution of 20 mM HEPES at pH 7.0 was used as a buffer for all dispersions. PoP solutions were prepared at varying concentrations (10 and 15 wt %) and stirred at room temperature for a minimum of 2 h to ensure complete dissolution of the protein. Calculations of the final protein concentrations were based on the actual protein concentration of the powder (∼90%). Sodium azide (0.02 wt %) was added to samples for bacteriostatic preservation.

### Preparation of Potato Protein Microgels

Microgel fabrication was based on the previous methodology of Sarkar et al.,^30^ Soltanahmadi et al.^31^ and Aery et al.^32^ Briefly, PoP solutions were thermally gelled by heating in a water bath at 80 °C for 30 min, followed by cooling in room temperature water for 10 min and refrigeration overnight at 4 °C. The macroscopic protein gel was then diluted at 1:1 w/w ratio with HEPES buffer at pH 7.0 and sheared for 3 min at 12,500 rpm using a hand blender (Bosch MSM6B150GB, U.K.). The dispersion of gel fragments formed was degassed (Intertronics, Thinky ARE-250), with 1 min of mixing at 2,000 rpm and 1 min of defoaming at 2200 rpm. Samples were finally passed through a custom-made jet homogenizer (Jet Homogenizer, University of Leeds, U.K.) for 3 cycles at 300 bar. Considering the bulk “parent” gels as 100 vol %, post 1:1 dilution, the resultant potato protein microgel dispersions contained 50 vol % microgels. These dispersions are subsequently referred to as PoPM-*X*, with the “*X*” denoting the wt % PoP from which the ‘parent’ gel was formed.

### Small Angle X-ray Scattering (SAXS)

SAXS measurements were conducted using the SAXSpoint 5.0 (Anton Paar GmbH, Austria). The instrument was equipped with a laboratory-based Cu–Kα radiation X-ray source, providing a wavelength (λ) of 1.54 Å and a 2D PILATUS detector (Dectris AG, Baden, Switzerland). The sample to detector distance (SDD) was calibrated using a silver behenate standard prior to the measurements. All measurements were carried out at 320 mm SDD, providing an accessible q-range between 0.1 to 6 nm, where q is the scattering vector magnitude. The temperature was controlled at 25 °C. A beam stop free setup was utilized to measure the direct beam intensity (*I*_0_), facilitating subsequent data reduction and background subtraction.^33,34^ Samples were loaded into quartz glass capillary tubes with an outside diameter of 1.5 mm (Capillary Tube Supplies Ltd., Cornwall, U.K.) then vacuum sealed. Transmittance scanning lasted 90 s, while sets of 5 frame acquisitions were completed lasting 120 s per frame. Empty capillary and buffer scattering patterns were subtracted from the data.

For protein characterization, the pair distance distribution function (PDDF) was computed using PCG software (University of Graz, Austria), representing a histogram of pair distances within the particle (protein material), weighted by the electron density contrast relative to the solvent. The method implements an indirect Fourier transform to obtain the PDDF.^35,36^

### Force Volume Atomic Force Microscopy (AFM)

To assess the modulus of microgels, force volume atomic force microscopy was used in a fluid environment to generate maps of the modulus across the sample. Similarly to our previous study,^22^ PoPM dispersions were diluted to a protein concentration of 0.01 vol % and approximately 150 μL of diluted sample deposited onto new, clean silicon wafers. Samples were left for 10 min to adsorb to the surface, then “washed” a minimum of six times with HEPES buffer via buffer replacement with a pipette, ensuring that the sample was constantly hydrated. Samples were then transferred to a MultiMode 8 AFM equipped with a Bruker Nanoscope V controller. Thermally stabilized silicon nitride AFM cantilevers with a nominal tip radius of 20 nm (MLCT-BIO–DC, cantilever C, Bruker Probes, Camarillo, CA) were used within a fluid cell filled with HEPES buffer at pH 7.0.^12,37^

For premeasurement calibration, the deflection sensitivity of the probes were assessed using an initial ramp on clean silicon, while spring constants of the cantilever were measured using the thermal noise method and found to be approximately 0.01 N/m.^12^ Force volume maps were captured at a rate of 3 Hz and ramp size of 600 nm over scan areas of 3 x 3 μm, with 1024 samples per ramp. Individual force curves were extracted from the maps and processed using Bruker NanoScope Analysis software v3.0. The approach curves were baseline corrected using a linear fit, and the zero contact point was found by extrapolation of linearized curves (with the distance scale transformed to a power 3:2 scale) as shown previously by Aufderhorst-Roberts et al., in their work on nanomechanics of synthetic pNIPAM microgels.^12^ With the contact point offset to zero, the curves become a measurement of indentation. Each indentation curve was then analyzed using OriginPro 2024b to assess their gradients against the Hertz model, with the elastic modulus being the only free parameter^12^ and utilizing a Poisson’s ratio of 0.3.^38^ Sharp upward deviation from the Hertz model indicated interaction with the silicon substrate, so only the initial portion of the curve was fitted.

### Langmuir Trough Experiments

Langmuir trough compressions were conducted at an air–water (A-W) interface, using 20 mM HEPES buffer as the subphase to ensure a constant pH of 7.0. Since microgels were present in a diluted and hydrated state, it was assumed that their density was equal to that of pure water. Due to the aggregated nature of PoP we also consider this a particle, with a density of 1.35 gcm^–3^.^39^ From the mean particle diameters obtained via dynamic light scattering (DLS) in Figure S1 (on average 160 and 240 nm for PoPM-10 and PoPM-15 respectively) and in our previous work,^22^ the volume of a particle can be calculated. Taking these particles as having the densities indicated above, the number of particles spread at the interface can be calculated assuming complete spreading of known volumes of dispersions of specific particle concentrations. This number was then divided by the trough area to give the mean area per particle (*A*_p_),^40,41^ which of course is an average value because there are actually a range of particle sizes in each dispersion. The use of *A*_p_ provides insight into potential particle behavior, however it must be noted that this method does not take into account that the exact size of PoP and PoPM may be altered due to their adsorption at the A-W interface and the application of compression.

To verify the behavior of the samples, two trough systems were utilized. First, the surface pressure (π) was acquired via a roughened mica Wilhelmy plate (*ca*. 5–6 cm in width), suspended from a force transducer (Maywood Instruments, Basingstoke, U.K.) at the center of a poly­(tetrafluoroethylene) (PTFE) diamond-shaped trough (maximum area 225 cm^2^).^40–42^ Diluted microgel dispersions were spread at the A-W interface using a 100 μL syringe; drops were slowly formed at the syringe tip and lowered to the A-W interface, the tip raised and protocol repeated. The Langmuir trough barriers then compressed the film symmetrically at a constant, very low rate of relative area decrease, such that the π-A_p_ curves were believed to be equilibrium isotherms.^42,43^

Additionally, a KN2002 Medium Langmuir–Blodgett trough (KSV NIMA/Biolin Scientific AB, Sweden) was used for depositions of monolayers. This trough had an area of 243 cm^2^ and filter paper Wilhelmy plates (10.3 mm in width, Biolin Scientific AB) were used. To produce isotherms of the monolayers, the barrier was moved at 15 mm min^–1^. Before spreading, for both troughs, the A-W interfacial area reduced to the smallest possible value and a vacuum line used to suck away any surface impurities and ensure π was <0.1 m Nm^1–^ before rapidly re-expanding the trough to its maximum area and spreading the sample. Spreading took 1–2 min and was followed by a delay of 10 min before film compression was commenced, to ensure that the film started in an equilibrated state. For further analysis of Langmuir isotherms values of a compression modulus (*E*_G_) were calculated as described by Tatry et al. (2022)^7^—see eq 1 below. Polynomial fits of *E*_G_ versus π were generated using OriginPro 2024b.

1 $$E_{G}=-\frac{d\pi }{d\ln A}$$

### Monolayer Imaging via Atomic Force Microscopy (AFM)

For deposition of monolayers, samples were compressed and held at a single target π for 15 min before being transferred via withdrawal through the A-W interface onto a mica substrate (area *ca*. 1 cm^2^) at a rate of 0.4 mm per minute. During monolayer transfer continuous slow adjustment of the Langmuir trough barriers via ensured that the desired π was maintained. The L-B films were then dried for a minimum of 24 h in a desiccator prior to imaging in air using TESPA-V2 AFM probes (Bruker Probes, Coventry U.K.) in tapping mode via a MultiMode 8 AFM equipped with a Bruker Nanoscope V controller. Images of 10 x 10 μm and 1 x 1 μm areas were analyzed at 512 samples per line using Bruker NanoScope Analysis v3.0. Other, continuous methods of deposition and compression,^80^ plus *in situ* pNIPAM-based monolayer structural characterization,^81^ have been used elsewhere, to collect different structures at a range of π corresponding to different positions on a single plate, which suggested distortion of structure when the films were air-dried and examined *ex situ*.

### Foam Analysis

Samples of PoP, PoPM-10 and PoPM-15 were diluted to 1 wt % protein content using HEPES buffer at pH 7. In 15 mL test tubes, 5 mL of each sample was subjected to 1 min of shearing at 15,000 rpm via rotor-stator homogenizer (Ultra-Turrax S25N-8G, IKA, Staufen) prior to microscopy. Light microscopy (Celestron PentaView LCD Digital microscope model #44348, California) was used to observe the samples with a 4× magnification lens. ImageJ software (version 1.53c, National Institute of Health, Bethesda) was then used to determine the size distribution of air bubbles and the mean bubble size was calculated from at least 100 bubbles in multiple images. Smaller bubbles (<20 μm) were counted independently and added to the distribution graphs. Confocal scanning laser microscopy (CLSM) was performed using a Zeiss LSM 880 inverted confocal microscope (Carl Zeiss MicroImaging GmbH, Jena, Germany). A stock solution of Fast Green (1 mg/mL) was used to stain the protein phase and excited at a wavelength of 633 nm to observe protein structure surrounding air bubbles.

### Statistics

SPSS statistical software (version 28) was used to identify the significant differences between the tested samples. For comparison between the two microgel samples an independent samples *t* test was utilized, while for analysis of more than two samples, a one-way ANOVA (Duncan test) was conducted. Significant differences were defined as when *p <* 0.05.

## Results and Discussion

### Small Angle X-ray Scattering (SAXS)

Figure 1 displays SAXS curves for samples of 10 wt % (Figure 1A) and 15 wt % (Figure 1B) PoP systems. SAXS measurements were conducted on samples of nongelled potato protein solutions (PoP), dispersions of microgels (PoPM) and bulk gels (labeled ‘Gel’, *i*.*e*. protein solutions gelled within the SAXS capillaries, subjected to the same time–temperature profile as the bulk gels described in the Methods above). For the microgel dispersions, the volume fraction of these systems was typically 50 vol %.^22^

**Figure 1:**
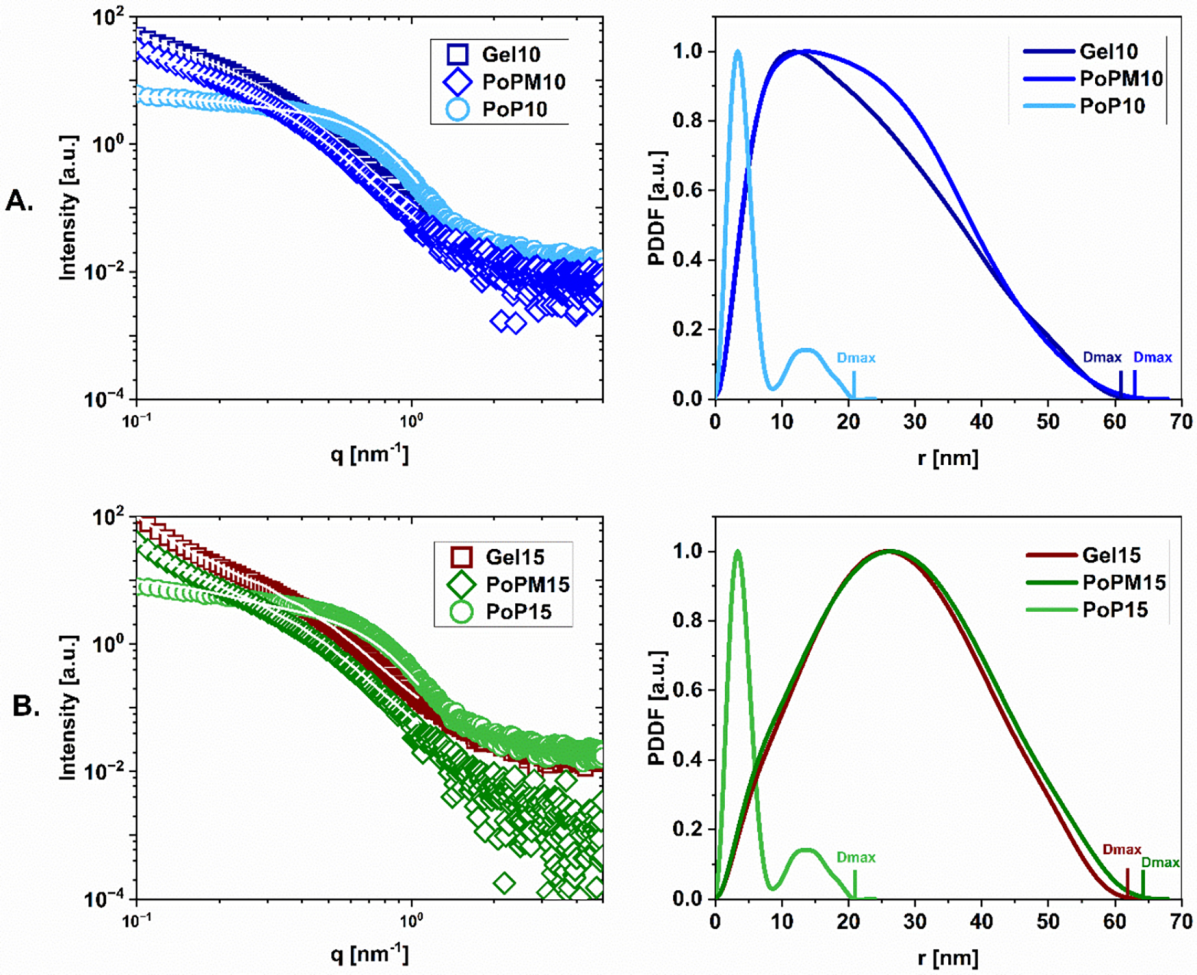
PoP samples at equivalent concentration formed ‘parent gels’ (squares), compared to nongelled protein solution (circles) and subsequently formed microgels in dispersion at 50 vol % (diamonds). The first column illustrates the SAXS profile while the second column displays Pair-Distance Distribution Function (PDDF) with respect to radial distance (nm). (A) shows systems associated with 10 wt % parent gels, while (B) displays samples of 15 wt % parent gels.

Figure 1 shows the change in scattering intensity from ungelled protein to gel and microgel samples. The changes in the slope of intensity between samples (see Figure 1) indicates changes in their structural characteristics. PoP in its ungelled state shows particle-like behavior, starting with a plateau at small angles followed by a decay in scattering intensity at higher scattering angles. When subjected to heating, the tertiary structure of PoP unfolds due to thermal denaturation and promotes the development of a dense gel network of noncovalent bonds with a small contribution from covalent disulfide interactions.^44^ Figure S2 illustrates this change in protein conformation via temperature-resolved SAXS measurements at temperatures increasing in 5 °C increments from 25 to 60 °C.

The pair-distance distribution function (PDDF) (see Figure 1) provides a quantitative understanding of size and shape changes of the studied structures. The PDDFs present distances inside particles and illustrate the evolution of size from protein in solution to larger nanoscale domains within microgels. The PDDF is characterized by a peak distribution function where the peak tail, and cutoff informs about particles size and shape.

The bimodal distribution within the PDDF of PoP at 25 °C suggests the presence of dimers and/or trimers (possibly originating from patatin and its dimers), which may become more prevalent when patatin is heated above its denaturation temperature^45,46^ (see Figure S2B). Within Figures 1 and S1, the bimodal distribution shown in the PDDF of PoP also suggests a dumbbell-like structure, which is comparable to prior studies.^47^ The dimensions of PoP gradually evolve with increasing temperature (demonstrated by increasing *D*_max_, where *D*_max_ is the cutoff in PDDF, equivalent to maximum dimension of the particle’s structure), reflecting its increasingly denatured and aggregated structure, see Figure 1.

Indeed, the maximum distances observed increase from 21 nm in PoP to 64 nm in microgel (PoPM-10 and PoPM-15) samples. We note that 60 nm is nearly the maximum observable limit of our SAXS experiments (*D*_obs_ = 2π/*q*_min_ (where *q*_min_ = 0.1 nm^–1^)) and hence, the existence of even larger structures should not be excluded. Furthermore, the radius of gyration (*R*_g_) displays an increase from 5.3 ± 1.6 nm in PoP to 22.1 ± 0.8 nm in PoPM-15 and 21.6 ± 0.5 nm in Gel15. The close values in *R*_g_ between the microgel and bulk gel samples highlight the similarity in their polymer networks. This was also observed for PoPM-10 and Gel10, which show *R*_g_ values of 18.6 ± 2.2 nm and 18.7 ± 2.4 nm, respectively. The most prominent structural changes appear to occur beyond 55 °C (see Figure S2) which corresponds to the denaturation temperature of patatin and onset of unfolding for α-helices and β sheets.^45^ This change in secondary structure was also reported in previous studies via Circular Dichroism.^22,32^ Furthermore, as shown in previous studies of whey protein microgels, heat denaturation promotes the formation of large collapsed domains in the gel structure,^48–50^ which also appears apparent for samples of potato protein ‘Gel’ and PoPM in Figure 1.

Assymetrically shaped PDDF plots are observed within Figure S2B (most significantly developed at 60 °C) as PoP solutions are heated, which can be associated with the elongation of the particles^51^ as protein polymers unfold. Subsequently PoP appears to adopt a more aggregated structure occupying larger distances which confirms the formation of the microgel particle systems observed for PoPM-10 and PoPM-15. Interestingly, in Figure 1 the PDDF of PoPM-15 shows a more symmetrical curve in comparison to the more asymmetrical PoPM-10 data (which instead appears to stretch toward larger dimmensions). This implies that the PoPM-10 sample is comprised of more cylindrical, elongated structures, while the PoPM-15 sample of greater concentration may be aggregated to a greater extent, yielding larger, spherical structures. Throughout heating and gel formation, two phenomena occur: protein aggregation and protein denaturation. Due to the higher concentration of protein within PoPM-15, perhaps this led to a more highly aggregated state thus preventing it from forming the elongated structures and altering the level of denaturation of proteins when compared to PoPM-10 samples. Further variables including cooling rate, pH and maximum temperatures would alter this behavior and should be studied in future work.

Microgel data within Figure 1 is also in accordance with our previous findings via DLS and AFM imaging^22^ that showed that, both PoPM-10 and PoPM-15 exist as aggregates; however PoPM-10 was found to be on average *ca*. 160 nm in diameter, while PoPM-15 exhibited a larger size *ca*. 240 nm in diameter (see Figure S1 of DLS size distributions). Although the light scattering within DLS assumes spherical particles and data are more weighted by the presence of larger particles.^52^ Conversely, SAXS offers a nanoscopic approach, focusing on small sized particles (i.e., < *D*_obs_) and hence, its combination with complementary techniques is necessary to visualize the structure of biopolymer systems across varying length scales.

### Force Volume Atomic Force Microscopy (AFM)

Force volume AFM mapping demonstrates the interaction force between an AFM tip and the sample surface in relation to the distance between them. This allows for the generation of a map of force curves across a sample area, which can then be used to measure the mechanical properties at the microgel particle level.^53^ This provides greater insight into the deformation of the biopolymeric microgel particles, rather than assuming that they have the homogeneous rheology of the parent bulk gel, since they are unlikely to have a constant modulus throughout their structure.^54^ As discussed above and in our previous work^22^ PoPM appear to exist at interfaces in an aggregated state, therefore our characterization of this sample aims to observe this unique structure in order to provide greater insight and enable its effective utilization within sustainable formulations.

Figure 2 displays Force Volume AFM height maps of samples taken from PoPM-10 and PoPM-15 in HEPES buffer at pH 7.0, plus a comparison of force curves taken from each of these samples. Both microgel samples exhibited a range of aggregate sizes and microgel particle fragments appear to be evident in both systems. This is particularly notable for the lower PoP concentration PoPM-10 (see Figure 2), likely due to the smaller size of the aggregates and thus lower levels of bonding. In contrast, the more concentrated PoPM-15 particles show a greater number of large aggregates. Therefore, to allow for sufficient comparison between the samples, curves for this Force Volume AFM analysis were selected on more central areas—which are likely to be aggregated clusters of more than one microgel particle. Figure 2 therefore shows aggregates which will be referred to as ‘particles’, and the corresponding force curves shown are taken from the center of these highlighted particles. Figure S3 displays the exact location of the force curves used in the analysis within Figure 2.

**Figure 2:**
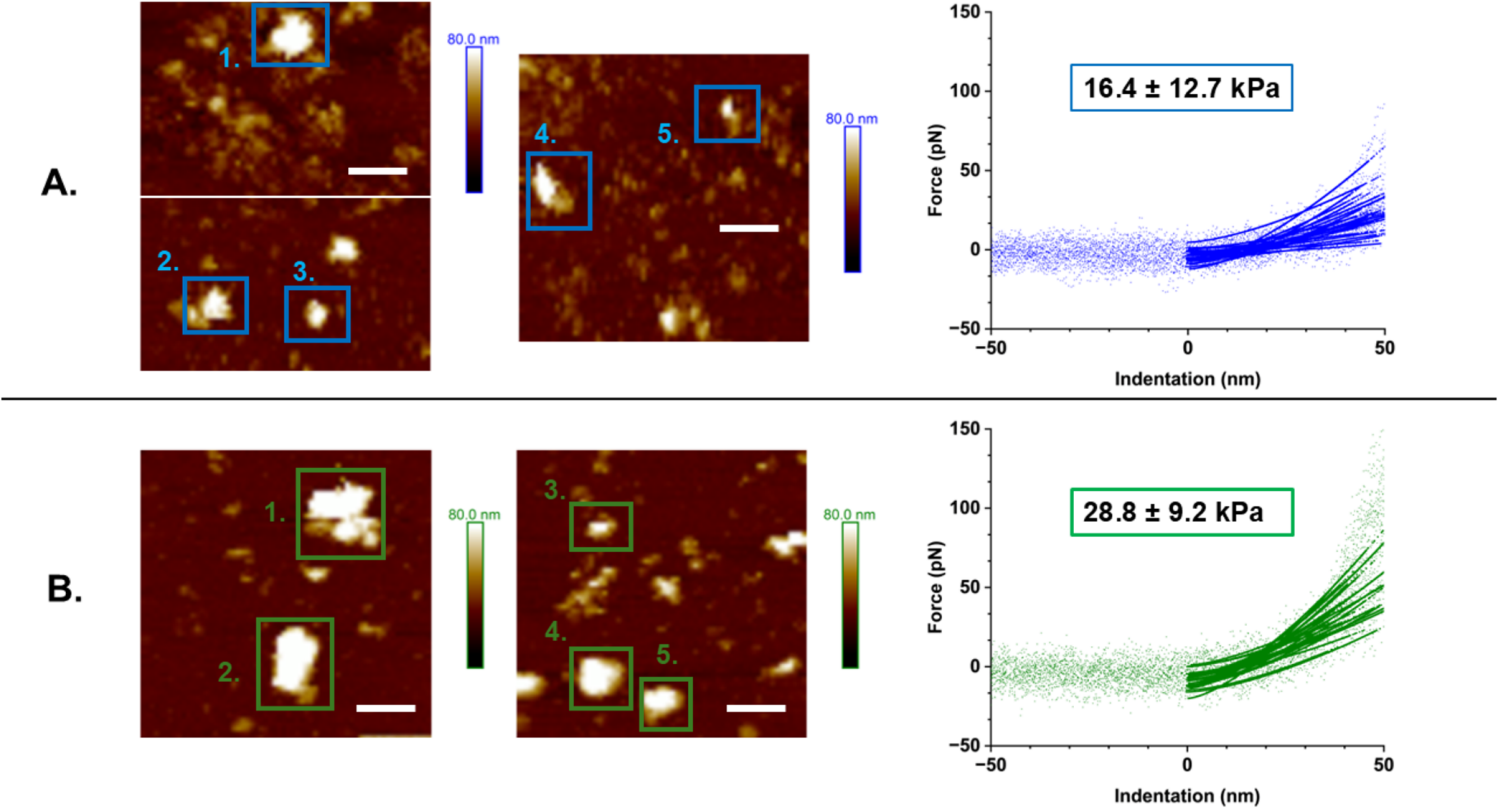
AFM height maps displaying particles used for measurements of force curves and the associated plots of force vs. indentation (dots) shown with corresponding Hertz model fits (solid lines). Data from 30 central curves taken from the outlined particles (labelled 1 to 5) was plotted for each microgel sample: (A) PoPM-10 (blue) and (B) PoPM-15 (green). The average modulus value calculated from these curves is shown for each sample, with different subscript letters indicating a statistically significant (*p <* 0.05) difference in values in Table S1. Scale bars on height maps =600 nm.

For each sample, 30 force curves were selected and fitted to a Hertzian contact mechanics model,^12^ as shown in Figure 2. The same curves are displayed on a wider scale (see Figure S4), illustrating the sharp increase in force seen when the probe reaches the stiff, silicon substrate. Overall, the average modulus values calculated from these indentation curves are significantly different: 16.4 kPa for PoPM-10 and 28.8 kPa for PoPM-15 (see Figure 2). Previously, the shear moduli of the bulk parent gels were measured via oscillatory rheology measurements, the gels being formed via heating in the rheometer.^22^ This yielded values in line with those within Figure 2: 20 kPa for PoPM-10 and 80 kPa for PoPM-15,^22^ although the Force Volume AFM-computed value for PoPM-15 particles is approximately a third of that for the bulk gel. The PoPM-10 and PoPM-15 particles present at the interface will have varying sizes^22^ and levels of aggregation, therefore this variation in the extent and height of clustering between samples is likely to have contributed to the differences observed between average moduli. Although the PoPM originate from a bulk parent gel, (and as noted above SAXS measurements observe very similar structures between microgel and bulk parent gel systems, see Figure 1), this gel itself will not be completely homogeneous and the protein within this network will exist in different structural states and levels of aggregation.^22^ The discrepancy between 15 wt % protein when observed as a bulk gel compared to AFM analysis of particles may be a reflection of this variation. Additionally, once exposed to the high-shear environment of homogenization during microgel fabrication, this gelled structure will fragment to differing extents, thus creating a system of protein particles which consist of a multitude of aggregated states and consequently varying moduli. However, these attributes are not often captured using traditional rheological approaches.^22^

Furthermore, for both samples, large values of standard deviation (*e*.*g*. in the range of 20 kPa) are seen when comparing moduli within the same particle: for comparisons between both central curves and those taken across particles see Tables S1 and S2. Both PoPM-10 and PoPM-15 are clearly inhomogeneous systems and this implies that all of the microgel particles exist with some areas of higher and lower moduli *e*.*g*. knots of aggregated protein created via the top-down method of their fabrication may exist across their structure^45^ (see Figures S3 and S4 and Tables S1 and S2).

Although values of modulus vary widely across potato protein microgel particles, the change in moduli does not appear to follow a gradient. Figure 3 displays force curves and their location on height and modulus force volume maps taken as transects across microgel particles for PoPM-10 and PoPM-15. This emphasizes the greater stiffness of the PoPM-15 particles compared to the PoPM-10, since the force curves for the former are clearly steeper. Moduli derived from these transect curves shown in Figure 3 also yield higher average values (when compared to central curves within Figure 2) of 26.4 and 52.6 kPa for PoPM-10 and PoPM-15 respectively. Overall, the average modulus for each sample (combining force curve data from central curves and transects *i*.*e*. data from Figures 2 and 3) is 21.4 for PoPM-10 and 41.6 for PoPM-15, which again confirms the greater stiffness of PoPM-15 systems.

**Figure 3:**
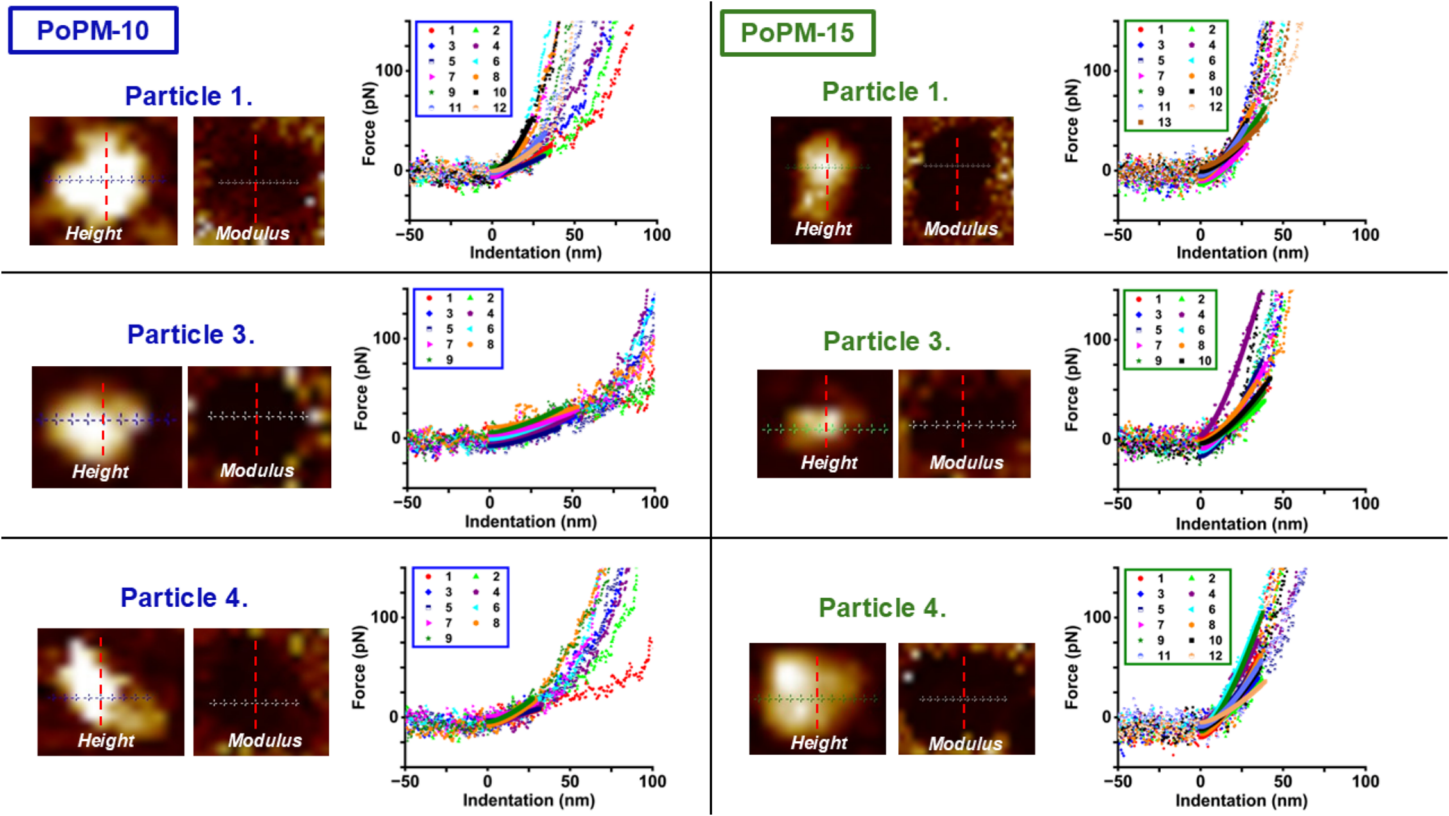
AFM height and modulus maps displaying individual particles (1, 3 and 4 in Figure 2) used for measurements of force curves and the associated plots of force vs. indentation (dots) shown with corresponding Hertz model fits (solid lines). Force curve data was taken along transects across the particles shown, with force curves right to left plotted chronologically in the adjacent graphs. Estimates of the center of particles are indicated with dashed red lines.

Figure 4 illustrates modulus values calculated from force curves within Figure 3 and shows a random array of moduli present across particles for both samples. Unlike the height of these regions, which generally decreases toward the periphery of the particle (Figure 3), this is not the case for the modulus values; instead, a range of moduli are observed across the particle regardless of their location (Figure 4 and Table S2). Conversely, studies of synthetic pNIPAM microgels have observed clear, smoothly varying elastic modulus gradients, which can be altered by changes to cross-linker type and concentration,^12,55,56^ but apparently this is not evident in microgels of protein origin.^14^ To our knowledge this is the first study of measurement of the modulus of plant protein-based microgels at an individual particle level. Whey protein microgel particles, also measured in a fluid environment at near-neutral pH, were also reported to have moduli in a similar range, of 12 kPa,^57^ however this whey protein work used lower protein concentrations than in the current study. With whey protein there is a greater prevalence of stronger disulfide bonds, which may be associated with more ordered gel structures.^45^ This is in contrast to the larger and denser aggregates of potato protein,^58^ which are reported to originate predominantly from hydrophobic interactions.^44^

**Figure 4:**
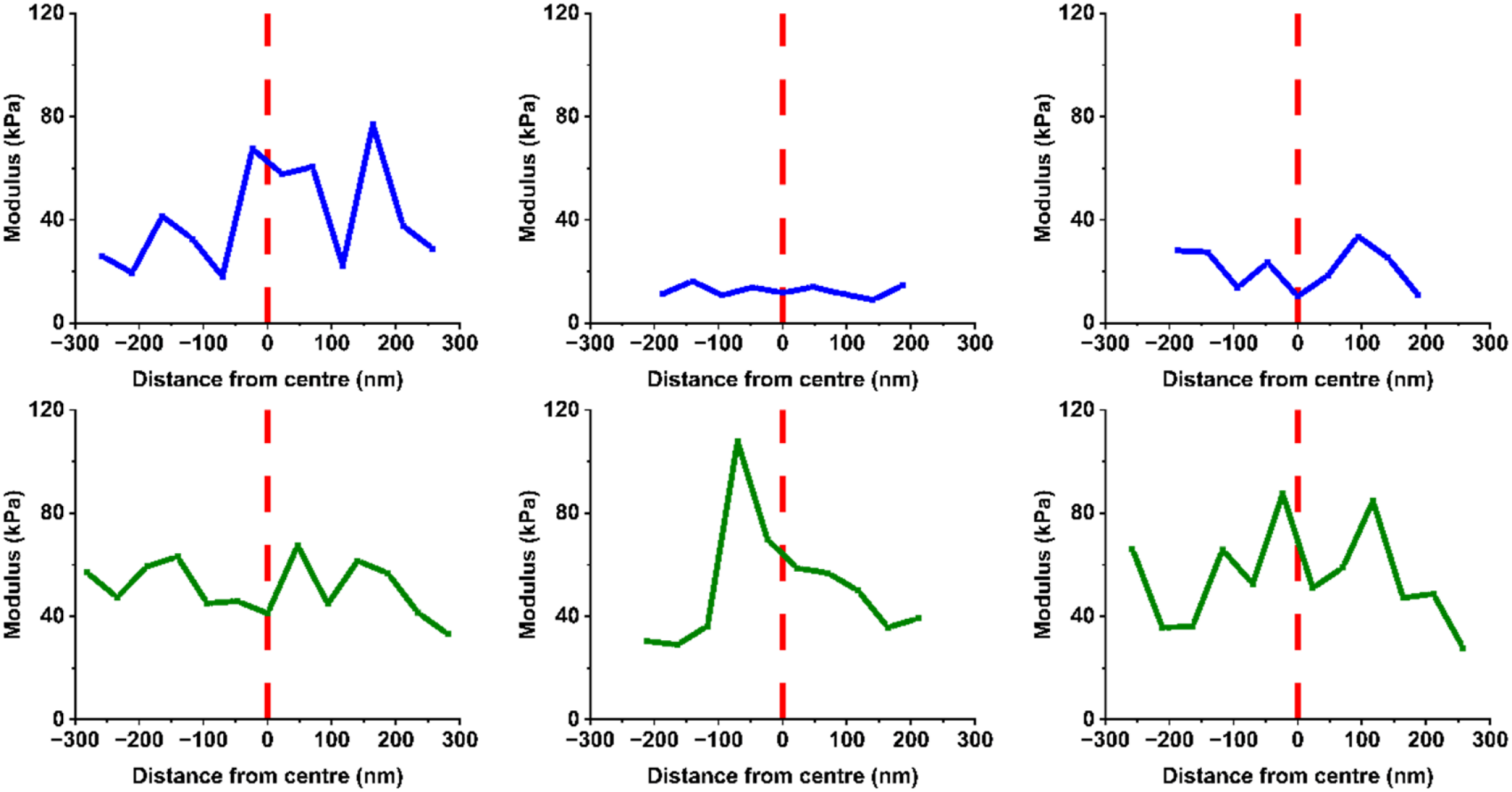
Plots of modulus vs. distance from the center of particles for each of the microgel particles in Figure 3 for PoPM-10 (blue, top row) and PoPM-15 (green, bottom row). In each case, modulus values at the estimated center of the particle are plotted at 0, with distances to the left of the center as negative values, while those to the right are shown as positive values.

### Monolayer Experiments

The π versus area per particle (*A*_p_) compression isotherms at the A-W interface of the PoP and PoPM provide an interesting comparison to our previous studies of interfacial shear rheology^22^ at the oil–water interface, not only due to the difference in interface type, but because the monolayer experiments involve spread particles rather than those adsorbed from solution. Previously, it was observed that adsorbed PoPM may show limited lateral interactions at the O–W interface, compared to PoP.^22^ Particles spread at high areas per particle are more likely to transform into an unfolded state compared to particles adsorbed from the bulk, which may diffuse to and persist at the interface in an aggregated form.^59^ Additionally, interfacial shear rheology monitors structure at constant area, while in Langmuir trough measurements the interfacial area is varied, which may act to facilitate interfacial rearrangement of particles.^60^

Figure 5 shows the increasing surface pressure (π) of monolayers of nongelled PoP, PoPM10 and PoPM15 under compression. All samples tested in this study reach a final π between 20 and 23 mN m^–1^, which suggests that a similar level of adsorption is reached regardless of microgelation,^61^ Figure S5 displays the standard deviation for completely separate spreading experiments for the 3 systems, demonstrating that there is good reproducubility of the spreading. The reproducibility of the compression isotherms (see Figure S5) and also their reversibility on repeated compression/expansion (see Figure S6) implies that there is little desorption of particles from the interface (at least up to these π), which attests to the relative ease with which such particles can be spread at the interface in the first place and is connected with their inherent surface activity, but also their larger size compared to when spreading protein molecules.^42,43^ We have also recalculated A_p_ taking into account the full vol % particle size distribution for each system (Figure S7) and the results are shown in Figure S8. This alters the absolute magnitude of the values of A_p_, because the smaller particles in the distribution are much more numerous, so that the separation of the ranges of *A*_p_ for PoP, POPM-10 and PoPM-15 are even larger, but not the relative positions of the isotherms, noting that *A*_p_ can only ever be an average for the whole film. Since the compressibility modulus (*E*_G_) refers to the gradient of the surface pressure *p* versus the *relative* change in area, via ln *A* in eq 1, using this alternative plot in Figure S8 to calculate *E*_G_, does not affect the *E*_G_ versus π plot (Figure 5B).

**Figure 5:**
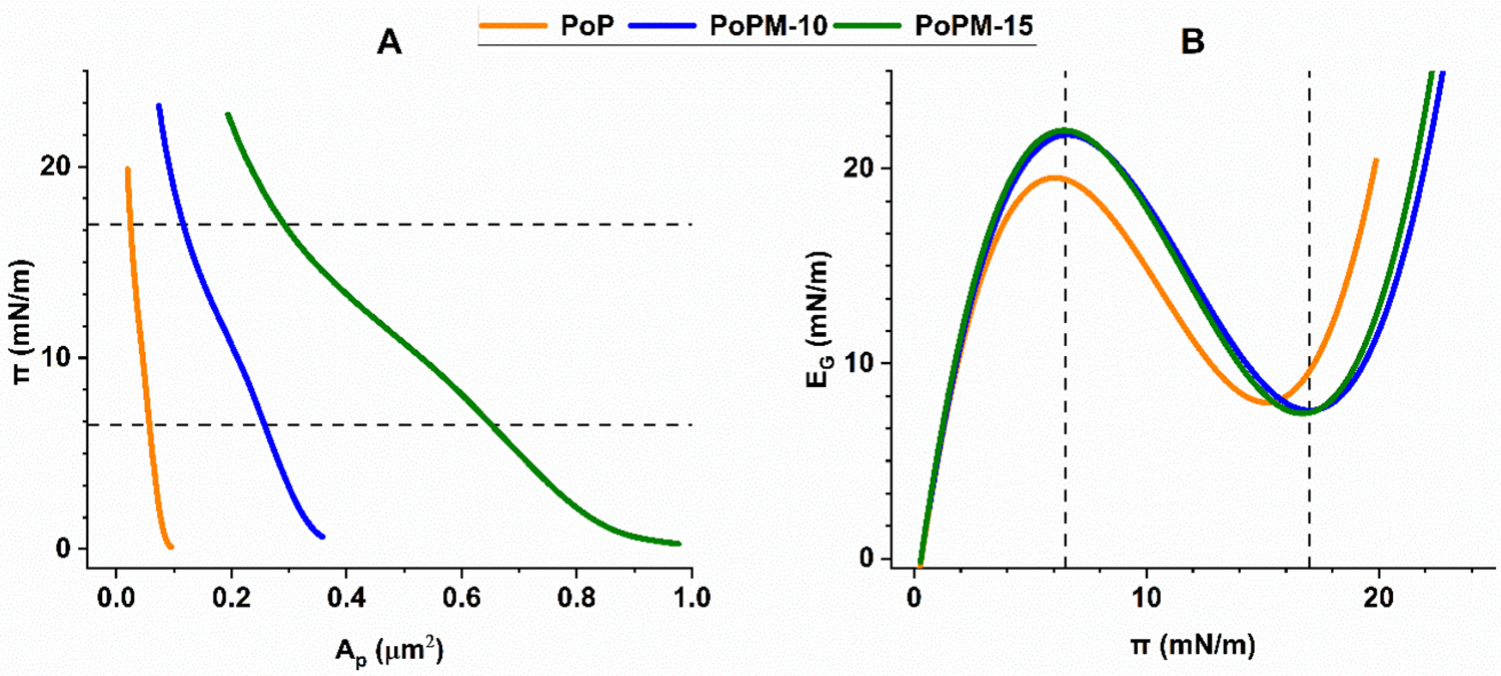
(A) Surface pressure (π) versus area per particle (*A*_p_) (μm^2^) compression isotherms at an A–W interface and (B) Compressibility modulus (*E*_*G*_) versus surface pressure (π) for samples of nongelled potato protein (orange), PoPM-10 (blue) and PoPM-15 (green). Dotted lines at surface pressures of 6.5 and 17 are included to highlight peaks and troughs in microgel compressibility. Isotherms are plotted based on triplicate measurements.

For monolayers of the larger, more concentrated PoPM-15 microgels, higher values of *A*_p_ and lower gradients of π vs. *A*_p_ are seen which implies that their compression is more gradual. Due to their larger size and higher protein concentration, it is likely that they are present in a more aggregated, stiffer form (as shown in previous AFM imaging^22^ and in Figure 2), thus their interaction and formation of lateral interactions at the interface may be restricted.^18^ Therefore, rather than forming a homogeneous layer, the PoPM-15 microgels may be present as clustered ‘islands’ across the interface. Nontheless, in all samples above *ca*. 3 mN m^–1^ a sharper increase in π is seen—see Figure 5A. This leads to a maxima in the compression moduli (*E*_G_)^7^ of all samples at *ca*. 6.5 mN m^–1^—see Figure 5B. These maxima may relate to the particles reaching a point of contact as they transition from a gaseous state to that of a liquid expanded film.^62,63^ However, the maximum appears to develop at a slightly lower π for the nongelled PoP, which may be a result of the PoP aggregates initially being in closer contact due to their smaller size and more flexible nature.^22^

It has been reported for synthetic microgels of defined core–corona structure, that the change in the elasticity and surface pressure of their monolayers with compression creates two maxima in *E*_G_: one associated with corona–corona interaction, the other at the point of contact between the rigid particle cores.^7^ More recently this model has also been associated with the storage globulin protein cupin, found in garden peas.^64^ However, as shown in Figures 3 and 4, it appears that these PoPM have a more random distribution of modulus across individual particles rather than defined regions. This might explain why the PoPM compression isotherms display only one apparent maxima in *E*_G_.

Compressing beyond this maxima in E_G_, where the compressibility decreases again (see Figure 5B), both microgel samples display a kink in their isotherms (at *ca*. 10 mN m^–1^ in Figure 5A), which suggests a change in monolayer configuration, perhaps as particles change conformation and/or lateral interactions develop between them.^65–67^ Finally a point of inflection can be seen in all samples, the gradient increasing in the order PoP > PoPM10 > PoPM15 (Figure 5A), which is again likely due to the greater flexibility of the nongelled PoP, asssociated with lower areas being occupied per particle.^68^ Overall this suggests that rather than the modulus of the microgel particles controlling their interfacial behavior, it is their size (both of individual particles and the aggregated forms in which they exist at interfaces) which largely dictates their capacity for interfacial packing. The inflection coincides with a minimum in *E*_G_ (Figure 5B), suggesting a final phase transition, perhaps as particles reach a maximum in their interaction and thus the compressibility also starts to recover (apparent as the sharp increase in *E*_G_ in Figure 5B).

As seen in Figure 5, all samples reached an equivalent final π, implying that all the monolayers studied reached a similar level of cohesion and molecular density,^69^ regardless of their size and composition. However, it was observed that values of π for PoP appeared higher than those of the microgel systems when studied using the trough that enables symmetrical compression^42^—see Figure S6 (rather that the uniaxial compressions reported in Figure 5). This may be a result of symmetrical compression promoting greater interactions between nongelled potato protein at the interface, reflecting similarity to our previous observations at the oil–water interface using interfacial rheology.^22^

It is notable that the π—*A*_p_ curves for all samples showed good reproducibility upon repeat compressions (Figure S6), presenting a similar shape across 3 cycles of compression (separated by high speed expansions back to the original maximum trough area used on initial spreading^40^). This suggests that attractive interactions within the monolayers of both nongelled protein and microgels are formed from completely reversible bonds, probably mainly hydrophobic bonding, which dissociate on removal of the applied stress. Furthermore, this indicates that although the monolayers are compressed there is no formation of irreversible aggregates,^69^ the materials retain their original stucture and are not displaced into the subphase.^40^ Thus, PoP, whether nongelled or in microgel form, seems adaptable to changes in interfacial compression and expansion, which could prove beneficial in the stabilization of foams (and probably emulsions as well).

It was hoped that more information on the structure of the monolayers would be revealed by the AFM imaging of the corresponding L-B films, while recognizing that the transfer process itself, but also the drying out of the films before examination, could have an influence on the structure of samples.^70^ Capillary forces^70^ may also contribute to microgel particle breakdown and the disturbance of noncovalent bonds between particles, as shown by Murphy et al.^71^ for whey protein microgels of a comparable size to those within this study. However, this behavior was also attributed to the low initial surface loading of samples, and microgels of a larger size were suggested to exhibit more resistance to breakdown.^71^

Figure 6 illustrates that all samples deposited at π = 18 mN m^–1^ show jammed structures where a background layer of protein is evident. SAXS confirms the formation of gelled particles via the structural differences observed between nongelled PoP and microgel dispersions (shown in Figure 1). However, microgel samples are mixed systems of microgel particles with the additional presence of microgel fragments as well as free or aggregated nonmicrogelled proteins. These impurities have also been reported in studies of gelled particles derived from whey protein.^14,21,72^ At such high π dense and relatively inert microgels will be forced to form bonds with neighboring particles, creating the tightly packed layers shown in Figure 6. This more cohesive structure still contains larger aggregates of microgel particles, however, compression is likely to have facilitated the formation of lateral interactions and thus creating a more viscoelastic interfacial layer.^13^

**Figure 6:**
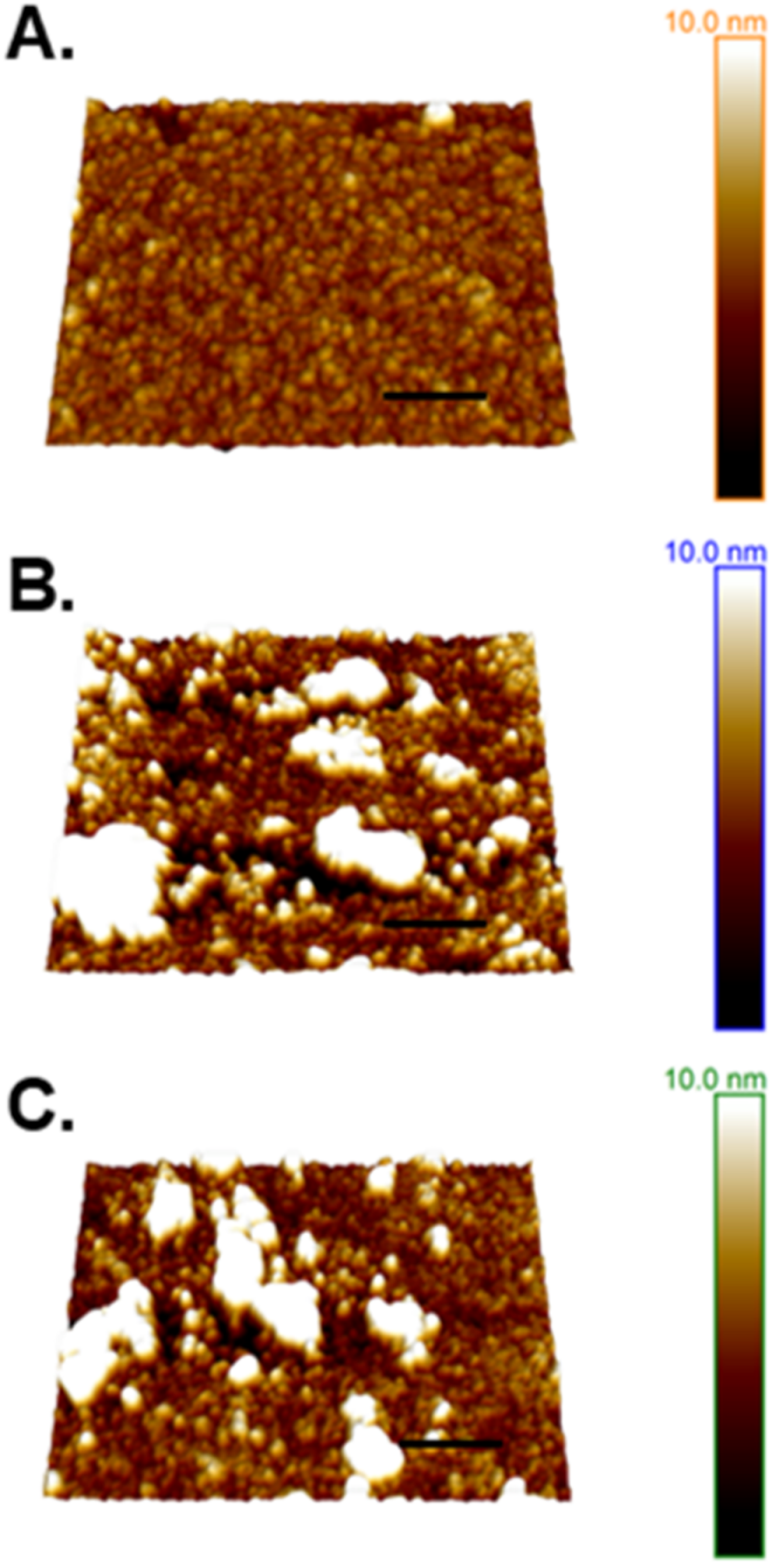
Topographic height images of samples deposited from the A-W interface via Langmuir–Blodgett transfer. Interfacial samples were compressed to a surface pressure of 18 mN m^–1^ before being transferred onto a mica substrate. From top to bottom, samples are (A) nongelled protein, (B) PoPM-10 and (C) PoPM-15. Scale bars = 200 nm.

Both PoPM-10 and PoPM-15 microgel samples reached similar final values of π, which suggests that they show similar microgel-microgel interactions and adsorption once enough compression is applied (Figures 5 and S6), which was observed across separate measurements for each sample sample type (Figure S5). Although, the larger sized PoPM-15 may require greater forces to promote intermolecular interactions and form a stabilizing layer with sufficient viscoelasticity to confer stability. For example, this could occur during emulsification or foaming, on the other hand, this may instead confer greater resilience to the PoPM-15 during the high shear conditions of such processing environments. Thus, further investigations of the behavior of PoPM within foaming and emulsion production conditions are required. Exploration of L-B depositions to observe structures at further π values may also help to confirm the exact structural changes occurring at the interface.

Overall, despite the presence of small fragments and apparent similar compressibility for samples of both of protein and microgel, there is a difference between the size of the samples and thus the interfacial layer thickness. Larger microgel samples may require an initial force to enable them to interact more efficiently at the interface, compared to smaller ungelled PoP which occupy a smaller area and thus have a greater tendency for lateral interactions. As shown in the AFM force volume investigation (Figures 3–5), smaller PoPM-10 particles of lower concentration displayed significantly lower values of moduli. However, both PoPM-10 and PoPM-15 samples displayed a high variability in the strength of their particles both between different particles but also across the width of particles themselves (Figure 5). This may result in a similar level of interfacial deformation across the particle (rather than defined deformation associated with a core–corona architecture), which facilitates similar levels of compressibility and lateral interactions once exposed to sufficient compression to enable close contact between particles (Figure 6). Increasingly, the role of interactions normal to the interface are considered pivotal in the behavior of microgels as emulsion stabilizers,^20^ which is not surprising given that surface–surface interactions dominate colloidal stability. As discussed in previous literature, low molecular weight fragments influence surface pressure significantly, but alone these cannot not form the thick, mechanically strong layers observed for systems with gel particles.^13,21^ Consequently, we now conclude this study with a preliminary comparison of the foam-stabilizing capabilities of PoP versus PoPM.

### Foam Experiments

Figure 7 displays samples of PoP, PoPM-10 and PoPM-15 observed using both light and confocal microscopy. Although the average air bubble sizes appear similar, these values do not include the diameters of the smaller bubbles: these were counted independently and added to the distribution graphs (see Figure 7). Both light and confocal microscopy highlight the prevalence of smaller bubbles (<20 μm) in microgel-based samples. Small bubbles are likely to instantaneously disappear in samples of protein due to the formation of a thinner interfacial layer which collapses easily under the interfacial stresses of interbubble gas diffusion.^73^ However, within microgel samples, the presence of thicker interfacial layers is thought to contribute to maintaining bubble stability.^74^

**Figure 7:**
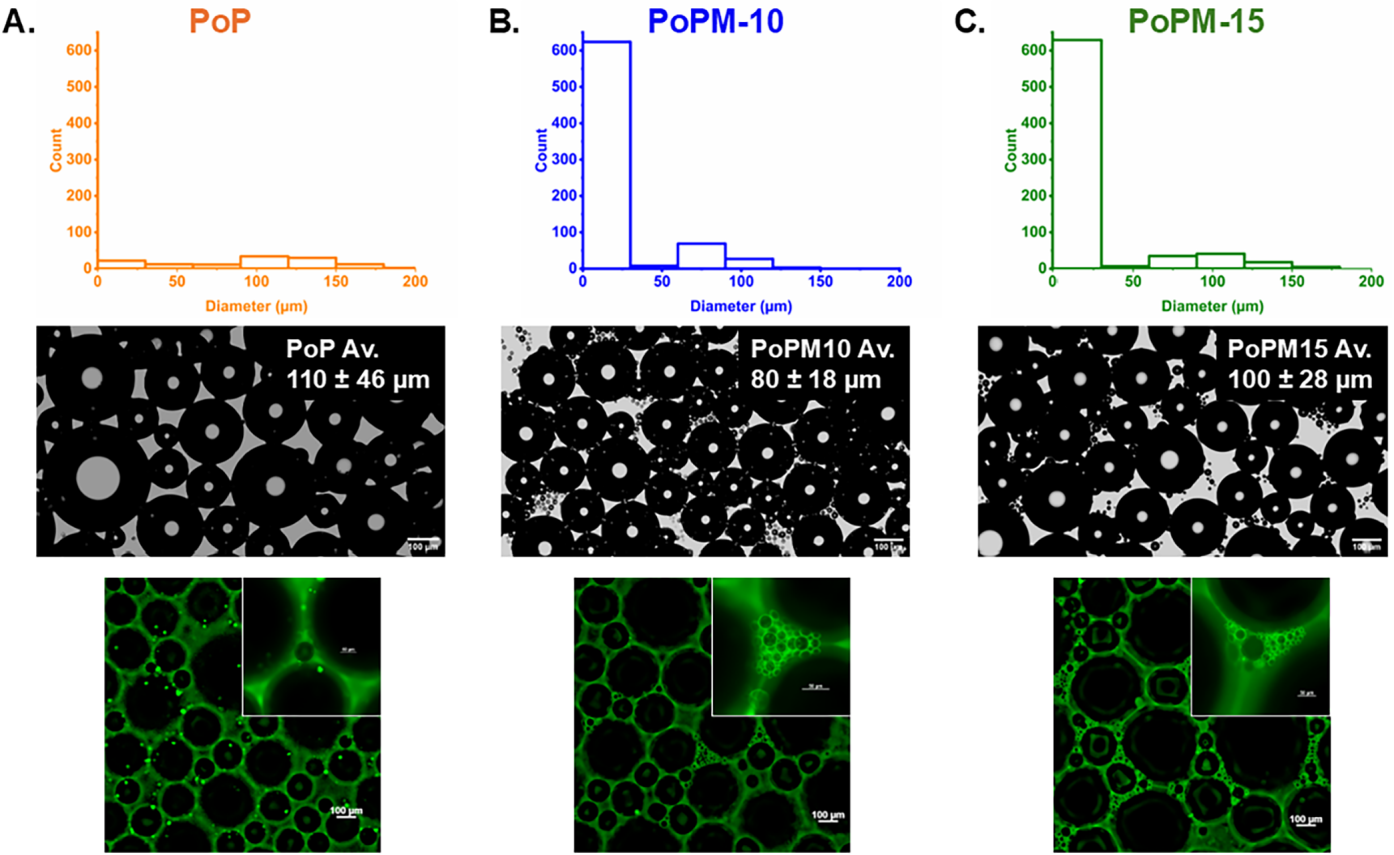
Analysis of air bubble size for samples of (A) ungelled PoP, (B) PoPM-10 and (C) PoPM-15. Histograms of air bubble size distributions and mean bubble size (top row) are based on light microscopy images (middle row), which highlight the presence of smaller bubbles of a few μm in diameter. Confocal microscopy (bottom row) illustrates the structure of these bubbles and the protein stabilizers. Size bars = 100 μm and 50 μm in the insets.

Small bubbles were also observed to persist at the boundaries of larger air bubbles (see Figure 7), adding a further means of stabilization against coalescence and disproportionation. Large or aggregated structures have been previously reported also to inhibit liquid drainage by blocking plateau borders, where they form a strong steric barrier to gas diffusion.^75,76^ It has been suggested that the presence of potato protein in an aggregated state may aid in promoting foam stability,^28^ implying that the presence of both aggregated protein particles and smaller bubbles may be beneficial in slowing foam drainage.^73^ Elsewhere, nongelled PoP reportedly forms stiff and more brittle interfacial layers;^77^ these may initially stabilize air bubbles but not offer stability against coarsening, leading to rapid shrinkage of bubbles in foams. Therefore, the use of a stabilizing microgel particle of a larger size than nongelled PoP may prove an effective tool in developing foam stabilizers. The implementation of alternatives to egg white proteins as foam stabilizers proves an ongoing challenge to mimic their functionality within food applications, while the high stability of PoPM may offer opportunities for greater stability of air bubbles to promote their use in improvement of product texture, color and novelty.^78,79^

Further investigation of microgel behavior over longer time scales and at O–W interfaces, i.e., relevant to O–W emulsions, will be undertaken in future work to provide further understanding of potato protein microgel behavior at real interfaces.

## Conclusion

This study has investigated the interfacial behavior of potato protein microgels compared to nonmicro-gelled potato protein at the air–water interface, particularly looking at nanoscale structures. SAXS confirmed the formation of microgels via alteration in protein internal structure upon heat treatment. Microgels formed from gels of a higher protein concentration displayed greater levels of aggregation, which may limit their development of lateral interactions at interfaces and thus lower the interfacial viscoelasticity that they impart.

Despite this, microgels of 10 and 15 wt % protein (PoPM-10 and PoPM-15) displayed an array of moduli across their structure when analyzed via force-volume AFM and reached comparable values of surface pressure to that of nongelled potato protein when spread monolayers of these materials were compressed at an A-W interface. Thus, we find for the first time that microgels have the capacity to reach a jammed interfacial state similar to that of nongelled potato protein and it is evident that the application of compression promotes intermolecular interactions between the microgel particles.

Potato protein microgels appear to be present at interfaces as large, densely packed, aggregated structures (in particular those formed from higher concentration gels) which may have limited lateral interactions at the interface until they are forced into close contact with their neighboring particles. Conversely, nongelled protein or smaller microgel fragments possess greater flexibility that enables them to expand to a greater extent at the interface, and in doing so spread out and interact with their neighbors. However, the thicker microgel-laden interfacial layers may have greater capacity to promote steric hindrance against coarsening, and offer greater mechanical resilience to environmental variation, as illustrated in their greater capacity to stabilize very small (<20 μm) air bubbles. Further investigations should consider the role of potato protein microgels as oil–water emulsion stabilizers.

## Supplementary Material



## Notes

Mean size distributions of PoP, PoPM-10 and PoPM-15 (Figure S1); SAXS measurements during heating in increments of 5 °C from 25 to 60 °C (Figure S2); force volume height and modulus maps with the position of the indentation curves used for data analysis (Figure S3); plots of force vs indentation shown for central curves with corresponding Hertz model fits against wider scales (Figure S4); moduli for 6 central curves from 5 microgel particles for each of PoPM-10 and PoPM-15 samples (Table S1); moduli for curves across 3 particles for each of PoPM-10 and PoPM-15 samples (Table S2); surface pressure vs. area per particle spread at the A-W interface in separate experiments, including standard deviation for triplicate measurements of systems of PoP, PoPM-10 and PoPM-15 (Figure S5); surface pressure vs. area per particle spread at the A-W interface for systems of PoP, PoPM-10 and PoPM-15 exposed to repeat compressions (Figure S6); vol % size distributions of PoP, PoPM-10 and PoPM-15 (Figure S7); surface pressure vs. area per particle of PoP, PoPM-10 and PoPM-15 using the vol % size distributions (Figure S8) (PDF)
