# Supplementary material for: Investigating the Interfacial Structure of Potato Protein Microgels at the Air–Water Interface
Source: Langmuir. 2025 Dec 16;41(51):34269–81. doi: 10.1021/acs.langmuir.5c04113 (PMC12756915; doi:10.1021/acs.langmuir.5c04113)
Supplement: Supplementary file 1 [file la5c04113_si_001.pdf]

Supporting Information

Investigating the interfacial structure of potato protein microgels at the air-water interface

Daisy Z. Akgonullu [1], Ryan Marr [2], Brent S. Murray [1,3] ***, Simon D. Connell [2]**, Amin Sadeghpour [1], Yuan Fang [3], Bruce Linter [4], Anwesha Sarkar [1,3]*

[1] Food Colloids and Bioprocessing Group, School of Food Science and Nutrition, University of Leeds, Leeds, LS2 9JT, UK

[2] Molecular and Nanoscale Physics Group, School of Physics and Astronomy, University of Leeds, Leeds, LS2 9JT, UK

[3] National Alternative Protein Innovation Centre (NAPIC), UK

[4] PepsiCo, Valhalla, New York, NY, 10595, USA

[5] PepsiCo International Ltd, Leicester, LE4 1ET, UK

*E-mail: A.Sarkar@leeds.ac.uk; ** s.d.a.connell@leeds.ac.uk; *** b.s.murray@leeds.ac.uk

Number of pages: 10

Number of figures: 6

Number of tables: 2

Contents

Figure S1. Mean size distributions of PoP (orange), PoPM-10 (blue) and PoPM-15 (green). The inset (table) shows the corresponding mean hydrodynamic diameter (dH) and polydispersity (PDI). Different superscript letters (a−c) indicate significant ($p < 0.05$) differences between polydispersity and PDI values....3

Figure S2. SAXS measurements during heating in increments of 5 °C from 25 °C to 60 °C for **A)** SAXS profile illustrating gelation of potato protein solution. Arrow indicates change in q with increasing temperature. **B)** Pair-Distance Distribution Function (PDDF) with respect to radial distance (nm) further observing potato protein gelation....4

Figure S3. Force volume height and modulus maps obtained using atomic force microscopy (AFM) with the position of the indentation curves used for data analysis marked across 5 particles each for samples of **A)** PoPM-10 and **B)** PoPM-15....5

Figure S4. Plots of force vs. indentation (dotted lines) shown for **A)** PoPM-10 (blue, left-hand side) and **B)** and PoPM-15 (green, right-hand side) with corresponding Hertz model fits (solid lines) against wider scales up to 100 nm indentation and 500 pN force. Data from 30 central curves is shown for each microgel samples. 6

Table S1. Moduli for 6 central curves from 5 microgel particles for each of PoPM-10 and PoPM-15 samples. Average and standard deviation (S.D.) of moduli for each microgel are displayed and different superscript letters (a-c) indicate significant ($p < 0.05$) differences between particles. 6

Table S2. Moduli for curves across 3 microgel particles for each of PoPM-10 and PoPM-15 samples. Force curve data was taken as transects across the particles shown in **Figure 3**, with force curves right to listed chronologically. Average and standard deviation (S.D.) of moduli for each microgel are displayed and different superscript letters (a-c) indicate significant ($p < 0.05$) differences between particles. 8

Figure S5. Surface pressure *vs.* area per particle spread at the A−W interface for systems of non-gelled potato protein (orange), PoPM-10 (blue) and PoPM-15 (green). Data are shown for triplicates of each sample to demonstrate the range in surface pressure which is shown through standard deviation of these data which is plotted as an overlay on top of the average of each sample in the corresponding colour of a lighter shade....9

Figure S6. Surface pressure *vs.* area per particle spread at the A−W interface for systems of non-gelled potato protein (orange), PoPM-10 (blue) and PoPM-15 (green). Data are shown for each sample when exposed to repeat compressions (“1st/ 2nd/3rd”) with quick expansions of the Langmuir trough between each compression. 10

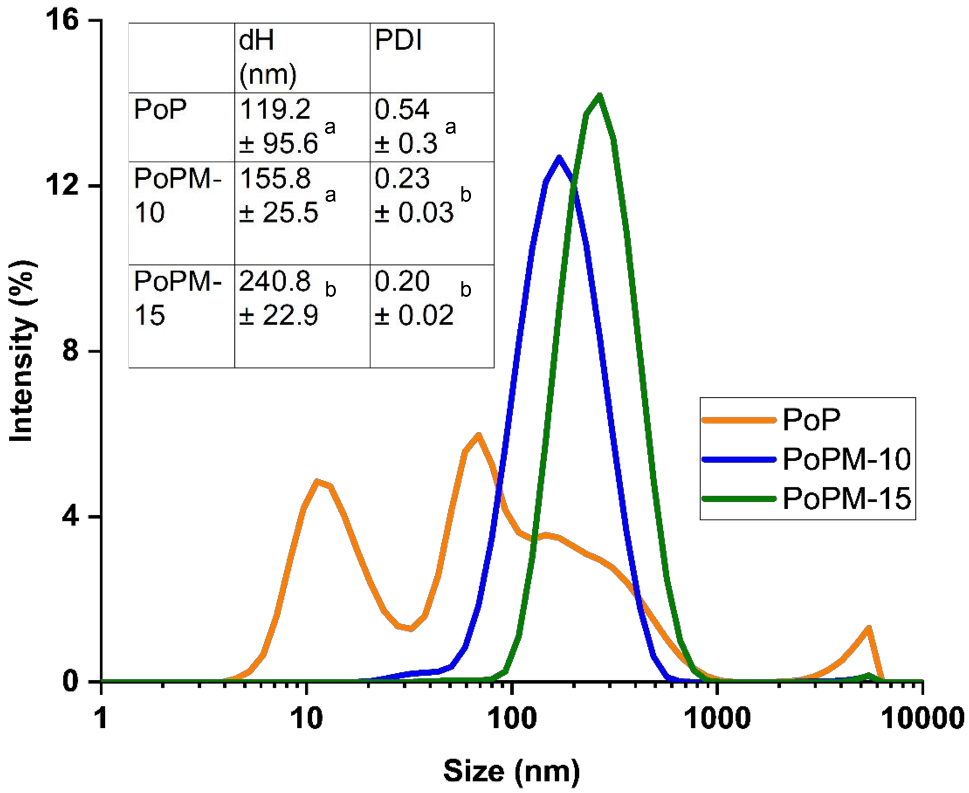

	dH (nm)	PDI
PoP	119.2 ± 95.6 [a]	0.54 ± 0.3 [a]
PoPM-10	155.8 ± 25.5 [a]	0.23 ± 0.03 [b]
PoPM-15	240.8 ± 22.9 [b]	0.20 ± 0.02 [b]

Figure S1. Mean size distributions of PoP (orange), PoPM-10 (blue) and PoPM-15 (green). The inset (table) shows the corresponding mean hydrodynamic diameter (dH) and polydispersity (PDI). Different superscript letters (a−c) indicate significant ($p < 0.05$) differences between polydispersity and PDI values

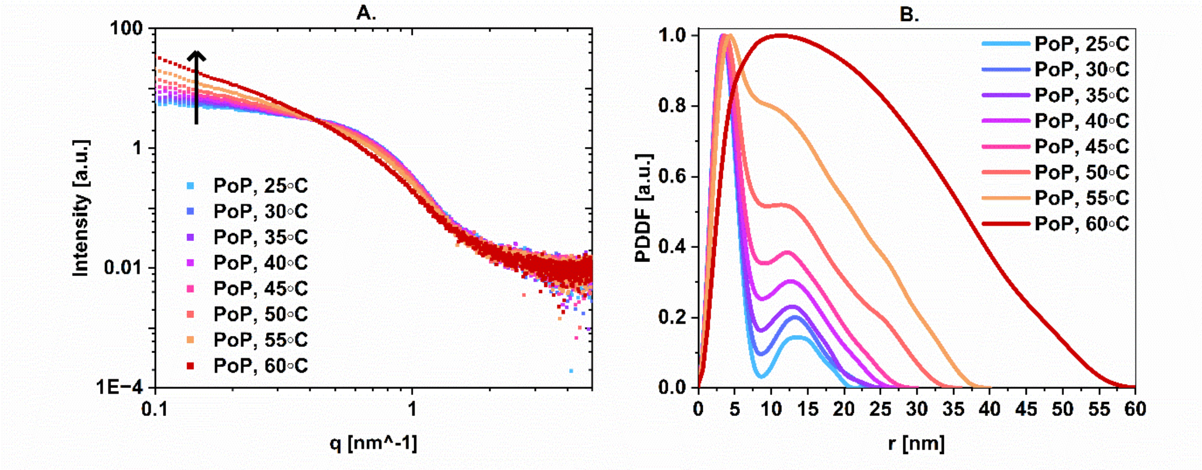

Figure S2. SAXS measurements during heating in increments of 5 °C from 25 °C to 60 °C for **A)** SAXS profile illustrating gelation of potato protein solution. Arrow indicates change in q with increasing temperature. **B)** Pair-Distance Distribution Function (PDDF) with respect to radial distance (nm) further observing potato protein gelation.

A)

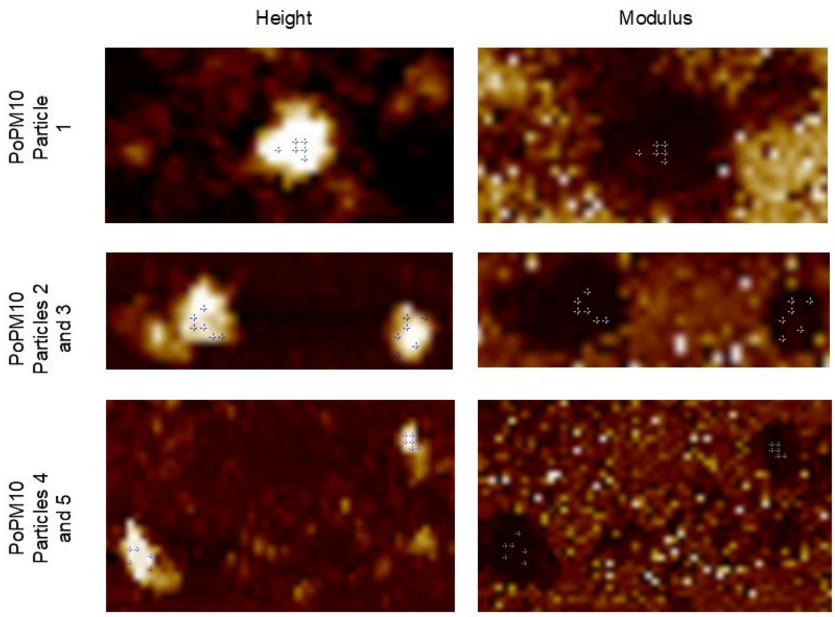

B)

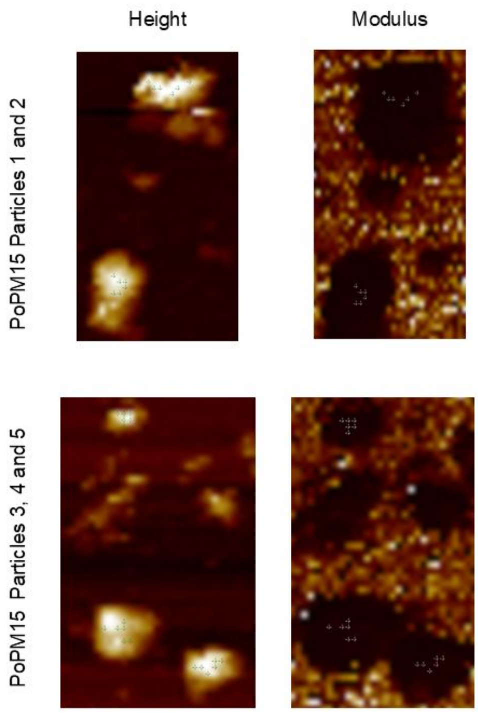

Figure S3. Force volume height and modulus maps obtained using atomic force microscopy (AFM) with the position of the indentation curves used for data analysis marked across 5 particles each for samples of **A)** PoPM-10 and **B)** PoPM-15

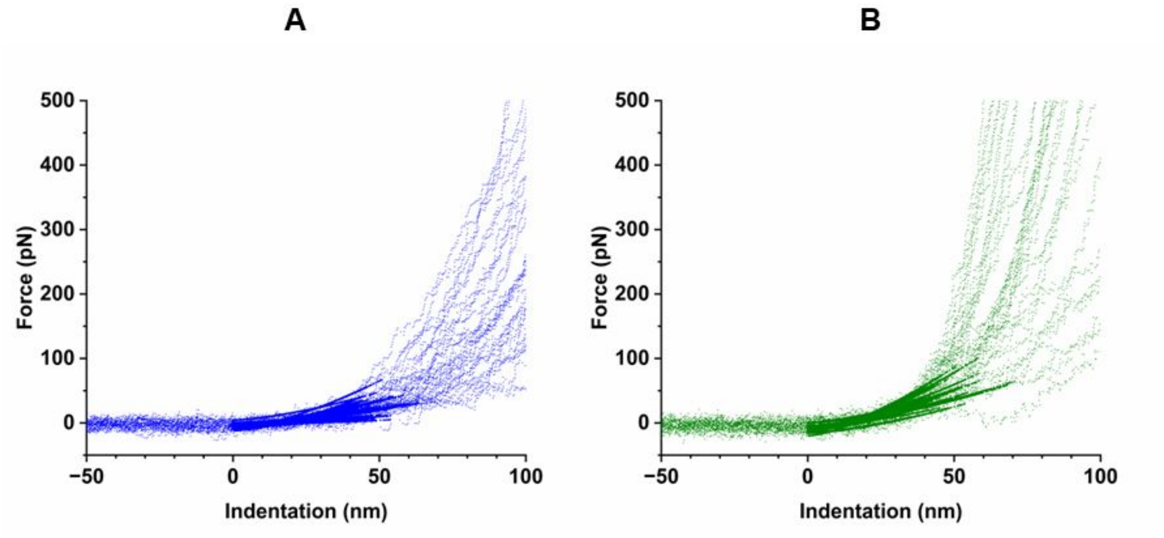

Figure S4. Plots of force vs. indentation (dotted lines) shown for **A)** PoPM-10 (blue, left-hand side) and **B)** and PoPM-15 (green, right-hand side) with corresponding Hertz model fits (solid lines) against wider scales up to 100 nm indentation and 500 pN force. Data from 30 central curves is shown for each microgel samples.

Table S1. Moduli for 6 central curves from 5 microgel particles for each of PoPM-10 and PoPM-15 samples. Average and standard deviation (S.D.) of moduli for each microgel are displayed and different superscript letters (a-c) indicate significant ($p < 0.05$) differences between particles.

	Particle 1	Particle 2	Particle 3	Particle 4	Particle 5
PoPM-10 Modulus Values (kPa)	3.43	9.64	9.86	13.34	3.20
	17.04	11.47	9.75	21.16	3.54
	6.59	13.45	12.48	60.38	15.11
	23.36	16.82	11.68	7.39	45.86
	10.61	18.38	12.06	12.38	30.27
	3.20	24.06	9.86	19.02	35.68
**Average**	10.70[a]	15.64[a]	10.95[a]	22.28[a]	22.28[a]
**S.D.**	8.08	5.25	1.26	19.30	17.70
	**Particle 1**	**Particle 2**	**Particle 3**	**Particle 4**	**Particle 5**
PoPM-15 Modulus Values (kPa)	23.47	20.92	51.25	32.14	15.78
	18.38	27.73	31.41	46.26	23.31
	18.95	19.11	20.77	31.62	31.15
	41.28	26.17	24.04	30.43	28.71
	18.43	29.02	20.15	41.69	25.85
	30.27	43.25	31.36	39.77	20.56
**Average**	27.70[a]	25.13[ab]	29.83[ab]	36.99[b]	24.23[a]
**S.D.**	8.55	9.15	11.60	6.50	5.59

Table S2. Moduli for curves across 3 microgel particles for each of PoPM-10 and PoPM-15 samples. Force curve data was taken as transects across the particles shown in **Figure 3**, with force curves right to listed chronologically. Average and standard deviation (S.D.) of moduli for each microgel are displayed and different superscript letters (a-c) indicate significant ($p < 0.05$) differences between particles.

Modulus (kPa)	PoPM10 Particle 1	PoPM10 Particle 3	PoPM10 Particle 4
**1**	25.99	11.25	28.02
**2**	19.45	16.18	27.49
**3**	41.42	10.72	13.77
**4**	32.63	13.88	23.41
**5**	18.06	11.68	10.34
**6**	67.49	13.93	18.43
**7**	57.70	11.20	33.59
**8**	60.49	9.00	25.29
**9**	22.29	14.52	10.93
**10**	77.05		
**11**	37.67		
**12**	28.77		
**Average**	40.75^{a}	12.48^{b}	21.25^{b}
**S.D.**	20.13	2.26	8.27
**Modulus (kPa)**	**PoPM15 Particle 1**	**PoPM15 Particle 3**	**PoPM15 Particle 4**
**1**	56.96	30.37	66.25
**2**	47.4	29.02	35.67
**3**	59.35	36.24	36.03
**4**	63.14	107.79	65.84
**5**	45.02	69.68	52.6
**6**	45.9	58.65	87.54
**7**	41.12	56.85	51.04
**8**	67.39	50	58.88
**9**	44.86	35.72	84.74
**10**	61.53	39.2	47.14
**11**	56.7		48.75
**12**	41.43		27.78
**13**	33.07		
**Average**	51.07^{a}	51.35^{a}	55.19^{a}
**S.D.**	10.36	23.96	18.58

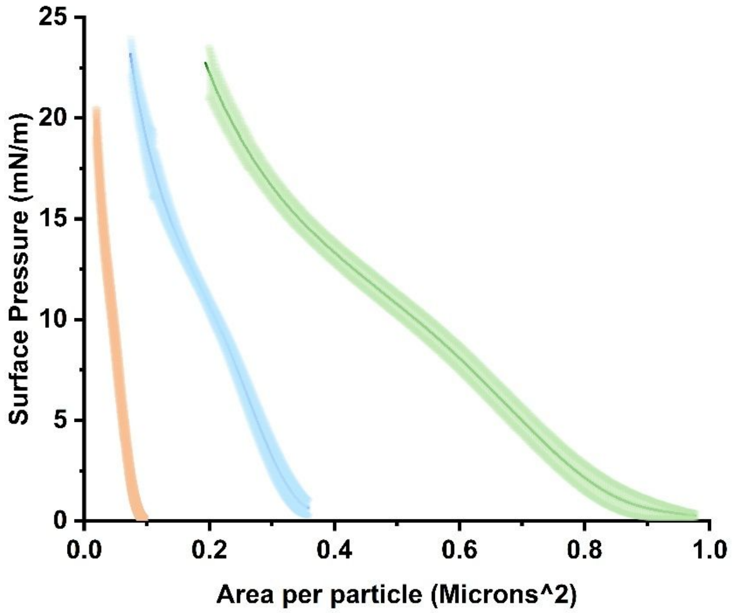

Figure S5. Surface pressure *vs.* area per particle spread at the A−W interface for systems of non-gelled potato protein (orange), PoPM-10 (blue) and PoPM-15 (green). The data from **Figure 5A** is shown with the addition of an overlay in the corresponding colour of a lighter shade which illustrates the standard deviation across triplicate measurements of surface pressure for each sample.

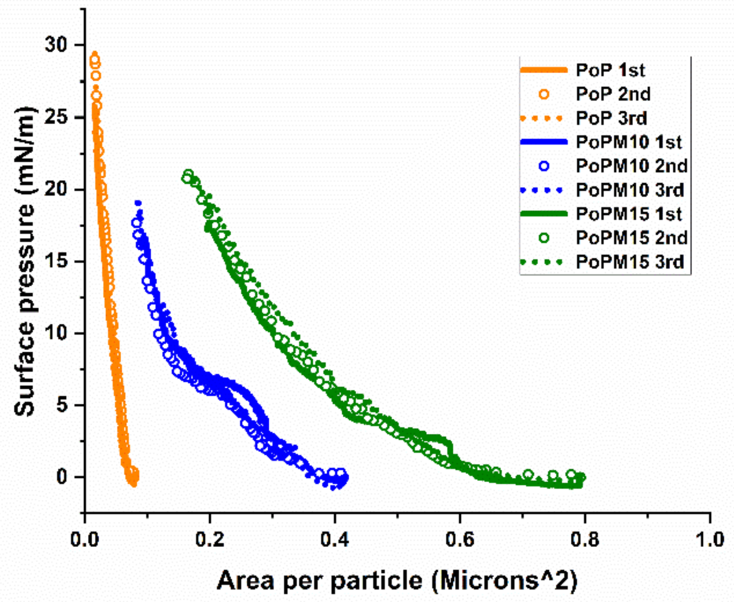

Figure S6. Surface pressure *vs.* area per particle spread at the A−W interface for systems of non-gelled potato protein (orange), PoPM-10 (blue) and PoPM-15 (green). Data are shown for each sample when exposed to repeat compressions ("1st/ 2nd/3rd") with quick expansions of the Langmuir trough between each compression.

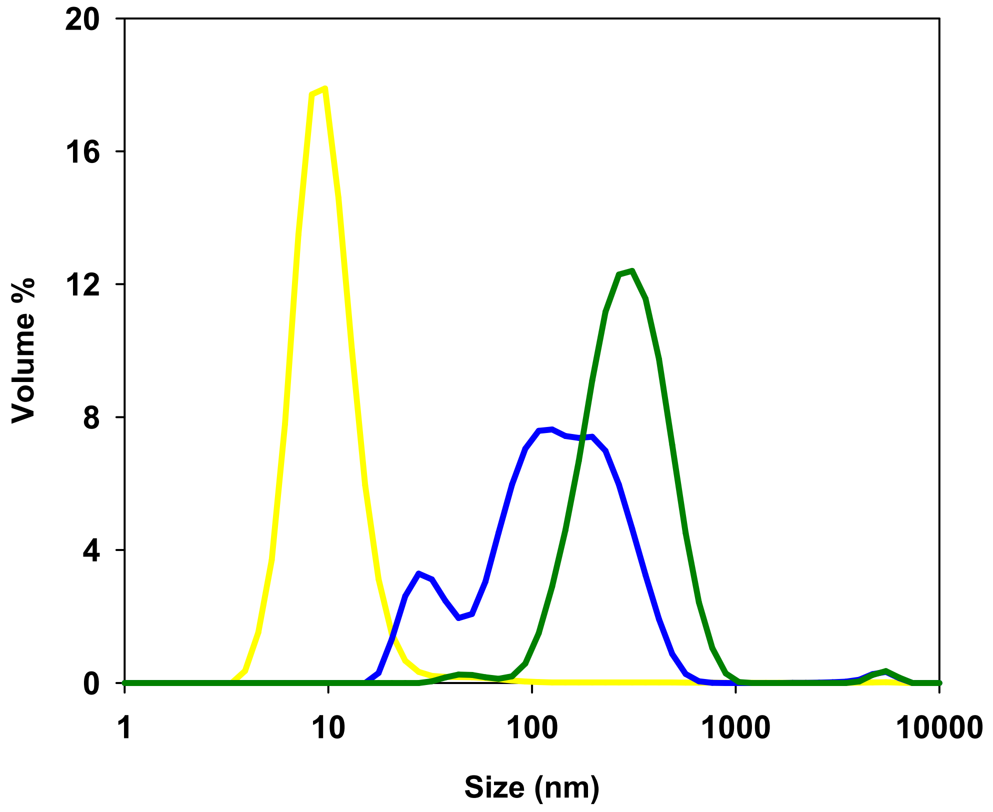

Figure S7. Vol% size distributions of PoP (yellow), PoPM-10 (blue) and PoPM-15 (green).

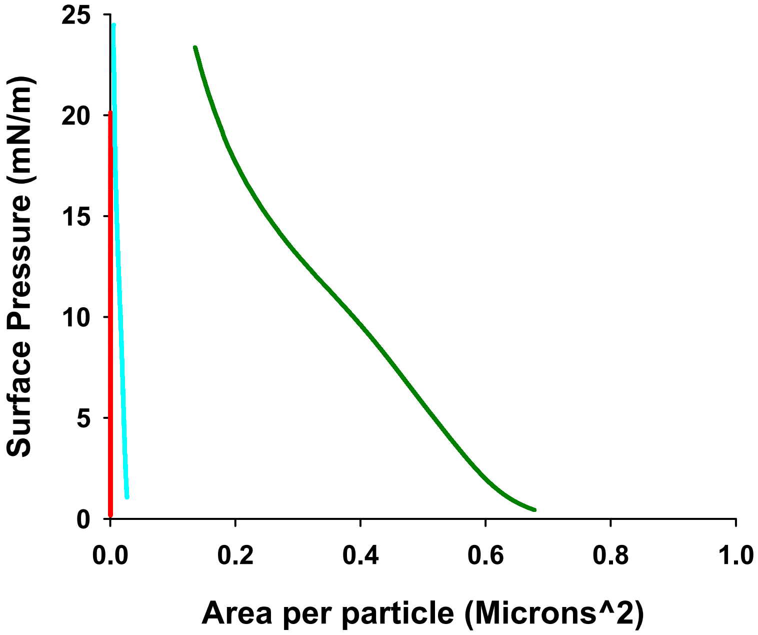

Figure S8. Surface pressure *vs.* area per particle spread at the A−W interface for systems of non-gelled potato protein (red), PoPM-10 (blue) and PoPM-15 (green) using the full vol% particle size distributions (Figure S7) to calculate the number of particle spread.
